# Managing Abnormal Uterine Bleeding in a Patient With Anti-Holley Antibodies and Tranexamic Acid Anaphylaxis: A Case Report

**DOI:** 10.7759/cureus.85559

**Published:** 2025-06-08

**Authors:** Waseem Khan

**Affiliations:** 1 Internal Medicine, Mercy Health St. Vincent Medical Center, Toledo, USA

**Keywords:** abnormal uterine bleeding, anti holley antibodies, blood transfusion, holley, tranexamic acid

## Abstract

Holley is an antigen of the Dombrock blood group system. Antibodies to Holley antigens, though extremely rare, if present, can result in hemolytic transfusion reactions. This poses a significant clinical challenge for clinicians, especially in patients who are actively bleeding and require blood transfusion. Thus, it is very important for clinicians to be aware of the presence of anti-Holley antibodies and address any management challenges while treating such patients. We report the case of a 47-year-old woman who had anti-Holley antibodies and presented with severe abnormal uterine bleeding (AUB). The patient had critical anemia with active bleeding and needed a blood transfusion, but the presence of anti-Holley antibodies made it challenging to find compatible blood for transfusion. Eventually, the patient underwent an emergency hysterectomy. To make the clinical situation further challenging, the patient developed an anaphylactic reaction to tranexamic acid. The patient was treated for anaphylaxis and subsequently stabilized. The patient was eventually discharged home in a stable condition.

## Introduction

Holley is one of the antigens of the Dombrock blood group system [1]. Multiple population-based studies have shown that the Holley antigen is present in 99-100% of the population [2], depending on the demographics studied. Therefore, it is very rare to harbor antibodies to the Holley antigen [2]. This, if present, can result in hemolytic reactions. These reactions can occur in association with blood transfusions. This poses a significant clinical challenge for clinicians, especially in patients who are actively bleeding and require blood transfusion. We present a case of a patient who had anti-Holly antibodies and presented with severe abnormal uterine bleeding (AUB). It is important for clinicians to be aware of the presence of anti-Holley antibodies and how to manage such challenging patients.

## Case presentation

A 47-year-old African American woman was transferred to our facility from an outlying hospital where she presented with AUB and severe anemia. The patient had a known history of AUB and had been followed by a gynecologist. She had tried different medications, which did not work for her. The patient also attempted scheduled nonsteroidal anti-inflammatory drugs (NSAIDs) without relief. A CT abdomen pelvis at that time revealed a normal-appearing uterus with endometrium measuring up to 2 cm.

This time the patient presented to the outlying hospital emergency department with complaints of worsening uterine bleeding. The patient reported changing a pad every 10-15 minutes and passing large clots. At the time of arrival at the emergency department, her hemoglobin was noted to be 8.2 g/dL, which decreased to 7.4 g/dL in the next three hours. The patient underwent a pelvic examination by an emergency doctor, which revealed active bleeding. A pelvic ultrasound was obtained and revealed a uterine size of 11 cm with fluid within the uterine cavity and an echogenic mass measuring 4.6 x 3.6 cm. The patient's prothrombin time was 14 seconds (11.5-14.2 seconds) with an international normalized ratio (INR) of 1.0. Fibrinogen was 319 mg/dL (203 mg/dL-521 mg/dL). Activated partial thromboplastin time (aPTT) was 28.2 seconds (23 seconds-36.5 seconds). The patient was given one dose of tranexamic acid intravenously and transferred to our facility (Table 1).

**Table 1:** Coagulation profile at the time of admission

Coagulation profile	Value	Reference range
Prothrombin time (seconds)	14	11.5-14.2
International normalized ratio (INR)	1	Not applicable
Activated partial thromboplastin time (seconds)	28.2	23-36.5
Fibrinogen (mg/dL)	319	203-521

Upon arrival at our facility, her bleeding had improved after receiving tranexamic acid; however, she reported that tranexamic acid made her feel lightheaded. She did not have any symptoms of anaphylaxis or angioedema at that time. The patient was admitted. Her vitals and hemoglobin levels were monitored. The patient again started having active bleeding the next day. She did have a drop in her hemoglobin to 6.5 g/dL. The trend of the patient's hemoglobin is given in Table 2 below.

**Table 2:** Hemoglobin trend during hospitalization

Hospital day	Hemoglobin level (g/dL)	Reference range (g/dL)	Clinical note
Day 0 (AM)	8.2	11.9-15.1	Hemoglobin on presentation
Day 0 (PM)	7.4	11.9-15.1	Hemoglobin after three hours
Day 1	6.5	11.9-15.1	Continued decline
Day 3	6.4	11.9-15.1	Hemoglobin post surgery
Day 4	7.3	11.9-15.1	Hemoglobin post transfusion

With the critically low hemoglobin, it was decided the patient would be given a blood transfusion. However, she was found to have anti-Holley antibodies, thus making it difficult to find compatible blood. This led to a challenging situation. Different methods were employed to minimize the need for transfusion, including judicious use of blood draws, iron transfusion, and administration of erythrocyte-stimulating agents while awaiting a compatible unit of blood [3]. Despite implying multiple medical measures, the patient's bleeding was not improving. After obtaining informed consent, the patient was taken to the operating room for a minimally invasive hysterectomy. Before the procedure, a decision was made to attempt to administer tranexamic acid once under anesthesia with a stable airway. Unfortunately, our patient did have an anaphylactic reaction to tranexamic acid in the operating room. It was immediately treated with epinephrine, hydrocortisone, and diphenhydramine [4,5].

Postoperatively, the patient was monitored. Lab draws were significantly reduced to absolutely necessary. She continued to have critically low hemoglobin. On postoperative day 2, the patient was able to receive a compatible packed red blood cell transfusion without any issues. The patient’s hemodynamics significantly improved, and she was eventually discharged home in a stable condition.

## Discussion

The Dombrock blood group system is made up of two antithetical antigens, a and b, and five high-prevalence antigens (Gy 9a0, Hy, Jo (a), DOYA, and DOMR) [1]. Holley is an antigen that is present in almost 99-100% of the population, depending on the demographics studied [2,6]. The Dombrock system was first identified in the 1960s [7]. The presence of antibodies to the Holley antigen is an extremely rare occurrence. But when it does occur, it poses significant clinical challenges. Since it is a very rare phenomenon, finding compatible blood for transfusion is extremely challenging. In most cases, there is usually a wait time of at least a few days before compatible blood can be sourced. This can result in significant morbidity and mortality, especially in patients with active bleeding or critically low hemoglobin. It is extremely important to be prepared for such circumstances.

There have also been a few case reports of safe transfusion of cryopreserved autologous blood collected at various stages during pregnancy [6]. However, there are no universal guidelines on these, mostly because of the rarity of these antibodies.

What makes our case report even more unique is that our patient developed an anaphylactic reaction to tranexamic acid. Tranexamic acid is usually used as an antifibrinolytic agent. It plays an important role in the management of patients presenting with uncontrolled AUB. However, there have been instances of anaphylaxis to tranexamic acid, in which case clinicians need to be prepared to find alternate means of bleeding control [4,5].

## Conclusions

Managing a patient with antibodies to the Holley antigen can be quite challenging. There is a paucity of data on the management of such patients. This is understandable because of the rarity of the condition. However, clinicians should be prepared and familiarize themselves with existing case reports and data regarding the management of patients with anti-Holey antibodies. These patients can be best managed with a multidisciplinary approach. Care should be undertaken to reduce blood draws to a minimum. Clinicians should also be prepared for unexpected scenarios, such as in the case of our patient, where she developed anaphylaxis to tranexamic acid. Clinicians should familiarize themselves with different innovative management techniques that have been employed for such scenarios in different parts of the world. Further studies are definitely needed to help design guidelines for the adequate management of such challenging patients.
